# Metabolic profiling of in vivo right ventricular function and exercise performance in pulmonary arterial hypertension

**DOI:** 10.1152/ajplung.00003.2023

**Published:** 2023-04-18

**Authors:** Catherine E. Simpson, Julie Coursen, Steven Hsu, Ethan K. Gough, Robert Harlan, Aurelie Roux, Susan Aja, David Graham, Matthew Kauffman, Karthik Suresh, Ryan J. Tedford, Todd M. Kolb, Stephen C. Mathai, Paul M. Hassoun, Rachel L. Damico

**Affiliations:** ^1^Division of Pulmonary and Critical Care Medicine, Johns Hopkins University School of Medicine, Baltimore, Maryland, United States; ^2^Department of Medicine, Johns Hopkins University School of Medicine, Baltimore, Maryland, United States; ^3^Division of Cardiology, Johns Hopkins University School of Medicine, Baltimore, Maryland, United States; ^4^Division of Human Nutrition, Johns Hopkins University School of Public Health, Baltimore, Maryland, United States; ^5^Molecular Determinants Core, Johns Hopkins All Children’s Hospital, St. Petersburg, Florida, United States; ^6^Division of Cardiology, Medical University of South Carolina, Charleston, South Carolina, United States

**Keywords:** metabolism, pulmonary hypertension, right ventricle

## Abstract

Right ventricular (RV) adaptation is the principal determinant of outcomes in pulmonary arterial hypertension (PAH), however, RV function is challenging to assess. RV responses to hemodynamic stressors are particularly difficult to interrogate without invasive testing. This study sought to identify metabolomic markers of in vivo right ventricular function and exercise performance in PAH. Consecutive subjects with PAH (*n* = 23) underwent rest and exercise right heart catheterization with multibeat pressure volume loop analysis. Pulmonary arterial blood was collected at rest and during exercise. Mass spectrometry-based targeted metabolomics were performed, and metabolic associations with hemodynamics and comprehensive measures of RV function were determined using sparse partial least squares regression. Metabolite profiles were compared with N-terminal prohormone of B-type natriuretic peptide (NT-proBNP) measurements for accuracy in modeling ventriculo-arterial parameters. Thirteen metabolites changed in abundance with exercise, including metabolites reflecting increased arginine bioavailability, precursors of catecholamine and nucleotide synthesis, and branched-chain amino acids. Higher resting arginine bioavailability predicted more favorable exercise hemodynamics and pressure-flow relationships. Subjects with more severe PAH augmented arginine bioavailability with exercise to a greater extent than subjects with less severe PAH. We identified relationships between kynurenine pathway metabolism and impaired ventriculo-arterial coupling, worse RV diastolic function, lower RV contractility, diminished RV contractility with exercise, and RV dilation with exercise. Metabolite profiles outperformed NT-proBNP in modeling RV contractility, diastolic function, and exercise performance. Specific metabolite profiles correspond to RV functional measurements only obtainable via invasive pressure-volume loop analysis and predict RV responses to exercise. Metabolic profiling may inform discovery of RV functional biomarkers.

**NEW & NOTEWORTHY** In this cohort of patients with pulmonary arterial hypertension (PAH), we investigate metabolomic associations with comprehensive right ventricular (RV) functional measurements derived from multibeat RV pressure-volume loop analysis. Our results show that tryptophan metabolism, particularly the kynurenine pathway, is linked to intrinsic RV function and PAH pathobiology. Findings also highlight the importance of arginine bioavailability in the cardiopulmonary system’s response to exercise stress. Metabolite profiles selected via unbiased analysis outperformed N-terminal prohormone of B-type natriuretic peptide (NT-proBNP) in predicting load-independent measures of RV function at rest and cardiopulmonary system performance under stress. Overall, this work suggests the potential for select metabolites to function as disease-specific biomarkers, offers insights into PAH pathobiology, and informs discovery of potentially targetable RV-centric pathways.

## Graphical abstract

**Figure:**
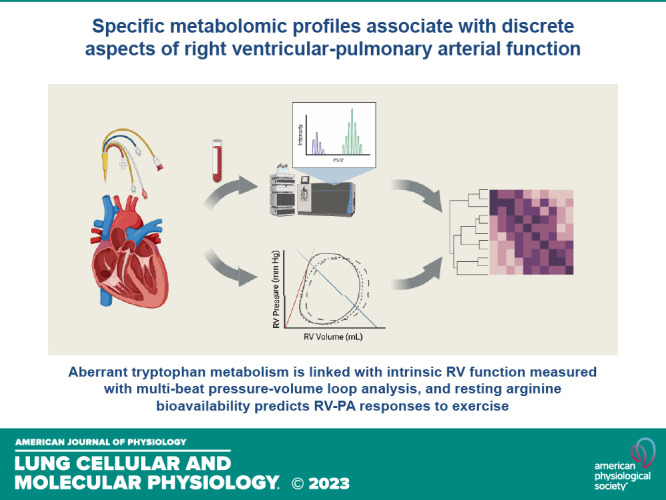

## INTRODUCTION

The status of the right ventricle (RV) is the major determinant of survival in pulmonary arterial hypertension (PAH; 1–3). Reliable surrogates of RV function would be valuable for predicting disease trajectory and assisting clinical management decisions, particularly if these markers are dynamic such that they vary as the RV changes. The N-terminal prohormone of B-type natriuretic peptide (NT-proBNP), the current clinical gold standard biomarker, is reflective of myocyte stretch and secreted by ventricular cardiomyocytes (4–8). As such, NT-proBNP is well-positioned to reflect pressure and volume loads presented to the ventricle, but it is perhaps less well-suited to reflect comprehensive RV function, particularly load-independent metrics such as contractility, ventricular relaxation, or measures of functional reserve (e.g., what will happen when the cardiopulmonary system is stressed).

PAH is characterized by dysregulated metabolism in the pulmonary vasculature and at the whole body level, and metabolomics can be used to characterize RV-PA dysfunction (9, 10). However, previous studies have examined metabolite associations with hemodynamics and the pressure-flow relationship, which are determined by characteristics of the pulmonary circulation, rather than the RV (9, 10). Cardiac-specific contributions to the cardiopulmonary unit can be characterized by examining pressure-volume relationships in the RV (PV loops; 3, 11). Invasive RV PV loops allow for gold-standard assessments of intrinsic RV contractility (end-systolic elastance, or Ees), ventricular diastolic function, and stroke work (12–14). Relating ventricular contractile function to effective arterial elastance (Ea), a lumped parameter reflective of afterload also derived from the PV loop, allows an assessment of how well contractile function is matched to afterload, a concept known as RV-PA coupling (Ees/Ea; 12, 15). When PV loops are measured at rest and with exercise, changes in RV contractility and chamber dilation illustrate RV functional reserve in vivo (13, 16).

The present study leverages targeted metabolomics to examine metabolic associations with rest and exercise hemodynamics, including PV loops at rest and with exercise, in a cohort of patients with PAH. Because exercise poses a stressor to the cardiopulmonary system that prompts PAH symptoms and can “unmask” occult pulmonary vascular disease, we sampled pulmonary arterial blood for metabolomics both at rest and during exercise. We sought to identify metabolic profiles that closely reflect RV functional parameters obtainable with PV loops and/or with exercise, hypothesizing that such profiles could outperform NT-proBNP in predicting RV function and exercise hemodynamics in PAH, and secondarily reveal fundamentals of underlying disease pathobiology.

## METHODS

### Cohort

Recruitment occurred at a single tertiary-care center through referrals for diagnosis or management of PAH. The study protocol was approved by the Johns Hopkins Institutional Review Board, and all patients gave written informed consent. Patients underwent cardiac magnetic resonance imaging (MRI), transthoracic echocardiography, right heart catheterization (RHC), and invasive RV PV loop analysis on the same day. Thereafter, patients were longitudinally followed for clinical worsening, which was defined by any one of: ≥ 10% decline in 6-min walk distance, worsening World Health Organization (WHO) functional class, PAH therapy escalation >3 mo after index RHC, RV failure hospitalization, or lung transplant or death.

### Hemodynamic Assessment

Patients underwent standard RHC with an eight French internal jugular introducer sheath, which was then exchanged for dual-entry nine French sheath for placement of five French PV conductance catheter and four French PA wedge catheter. The PV catheter was maintained in place during exertion, and peak exercise was defined as symptom-limited maximum effort with at least stage 2 (25 W, 4 min) exertion using supine cycle ergometry during RHC. Serum samples were collected from the pulmonary artery during rest and exercise. Multibeat PV loops were constructed based on simultaneous measurements of pressure and volume at different loading conditions, as previously described (12, 13). PV loops were analyzed to derive ventriculo-arterial measurements including Ees and Ea, with the ratio of elastances calculated as Ees/Ea. τ, a load-independent time constant of RV relaxation, was also calculated. Supplemental Fig. S1 in the Online Supplement outlines and describes the key ventriculo-arterial variables that were evaluated for metabolomic associations.

PAH was diagnosed by a mean pulmonary artery pressure (mPAP) ≥ 25 mmHg, pulmonary vascular resistance (PVR) ≥ 3 Wood units, and pulmonary artery wedge pressure (PAWP) ≤ 15 mmHg during RHC, which was the consensus definition at the time of cohort enrollment (17).

### Targeted Metabolomics

Multiplexed liquid chromatography-mass spectrometry-based targeted metabolomics were performed on patient plasma samples at the Johns Hopkins Molecular Determinants Core at All Children’s Hospital. All samples were obtained under fasting conditions. After standard sample preparation, high-pressure liquid chromatography was accomplished using a Shimadzu HPLC comprised of a SIL-30ACMP 6-MTP autosampler and Nexera LC-30AD HPLC Pumps (Shimadzu, Kyoto, Japan). Chromatographic separation was performed using a pentafluorophenylpropyl column. Mass spectrometry was performed using a triple quadrupole (QQQ) mass spectrometer (Shimadzu, LCMS-8060, Kyoto, Japan) equipped with an electrospray ionization source used in both positive and negative mode. Each batch of samples was run with a system suitability quality control, which was created from commercially available plasma. Two hundred and forty-one compounds plus 18 heavy standards were measured. Chromatographic integration was performed using LabSolutions Insight (v.3.5, Shimadzu, Kyoto, Japan).

### Statistical Analysis

Paired *t* tests were completed to assess for metabolite abundances that changed significantly from rest to exercise. Associations between metabolites and RV-PA clinical variables were examined using sparse partial least squares regression (sPLS) with the *spls* package for R (18). The optimal tuning parameter was selected by 10-fold cross validation. Metabolite and clinical data were rescaled by mean-centering and dividing by the standard deviation of each variable to implement sPLS and to facilitate interpretation and comparison. Pre-exercise, postexercise, and the difference in pre- and postexercise (delta exercise) metabolite measures were analyzed separately, and we fit a separate model for each cardiopulmonary measure. Logistic regression was used to examine metabolite associations with clinical outcomes. Significant metabolite predictors of clinical outcomes were selected by harsh criticism thresholding with the *fdrtool* package for R (19, 20). Model accuracy comparisons between metabolites and NT-proBNP were performed using the area under receiver operating characteristics curves (AUC) for binary outcome variables with the *ROCit* package for R, and R-squared between observed and predicted outcomes for continuous variables. Pathway enrichment analysis and pathway topology analysis were performed to contextualize metabolomics results at the metabolic pathway level, and pathway impact values were calculated using the *MetaboAnalystR* package for R (21). All analyses were performed using R Statistical Software (v.4.1.2; R Core Team 2021).

## RESULTS

Our PAH cohort (*n* = 23) was predominantly female (83%) and predominantly white (83%; Table 1). In general, subjects had mild-to-moderate disease, with median mPAP of 33 mmHg and PVR of 4.7 Wood Units. Median right ventricular ejection fraction (RVEF) assessed by cardiac MRI was preserved at 50% (interquartile range 39%–57%), however, the median RV-PA coupling ratio was less than 1.0 at 0.66 (IQR 0.45–0.99) implying decoupling of RV contractility from afterload. Sixteen subjects had systemic sclerosis-associated PAH (SSc-PAH), and seven subjects had idiopathic PAH (IPAH).

**Table 1.** Demographics and clinical characteristics

Variable	Median (IQR) or *n* (%), as appropriate
Age, yr	61.00 (47.00, 66.00)
Sex (% female)	
Female	19 (82.6%)
Male	4 (17.4%)
Race (% white)	
White	19 (82.6%)
non-White	4 (17.4%)
Subtype (% IPAH vs. SScPAH)	
IPAH	7 (30.4%)
SScPAH	16 (69.6%)
WHO FC, n I/II/III	
1	1 (4.3%)
2	11 (47.8%)
3	11 (47.8%)
pro-BNP, pg/dL	326.00 (116.00, 681.50)
Cr, mg/dL	0.90 (0.80, 1.00)
RAP, mmHg	7.00 (3.50, 10.00)
meanPAP, mmHg	33.00 (27.00, 47.00)
PAWP, mmHg	10.00 (6.00, 12.00)
CO, L/min	4.60 (4.20, 5.33)
PVR, Wood units	4.67 (2.86, 8.39)
V̇o_2peak_, mL/kg/min	10.50 (8.97, 12.43)
RER	0.93 (0.89, 1.00)
Ees (RV contractility)	0.52 (0.43, 0.69)
Ea (RV afterload)	0.69 (0.52, 1.08)
Ees/Ea (RV-PA coupling)	0.66 (0.45, 0.99)
RVEF, %	49.62 (38.53, 56.88)
τ Suga	32.28 (24.27, 37.66)
RV end diastolic volume, mL	177.63 (132.70, 196.95)
meanPAP at 25 W, mmHg	48.00 (39.00, 64.00)
PAWP at 25 W, mmHg	15.00 (9.00, 20.00)
CO at 25 W, L/min	9.30 (6.97, 10.00)
PVR at 25 W, Wood units	5.12 (3.56, 7.87)
mPAP/CO multipoint slope	4.64 (1.51, 7.01)
PAWP/CO multipoint slope	0.87 (0.03, 2.41)
Taking PDE5 inhibitor	
Yes	13 (56.5%)
No	10 (43.5%)
Taking ERA	
Yes	17 (73.9%)
No	6 (26.1%)
Taking prostanoid	
Yes	0 (0.0%)
No	23 (100.0%)

CO, cardiac output; Cr, creatinine; Ea, end-arterial elastance; Ees, end-systolic elastance; ERA, endothelin receptor antagonist; FC, functional classification; IPAH, idiopathic pulmonary arterial hypertension; IQR, interquartile range; meanPAP, mean pulmonary arterial pressure; PAWP, pulmonary artery wedge pressure; PDE5 inhibitor, phosphodiesterase-5 inhibitor; pro-BNP, brain-type natriuretic peptide pro-hormone; PVR, pulmonary vascular resistance; RAP, right atrial pressure; RER, respiratory equivalence ratio; RVEF, right ventricular ejection fraction; SSc-PAH, systemic sclerosis-associated pulmonary arterial hypertension; V̇o_2peak_, maximum oxygen uptake; WHO, World Health Organization.

### Rest-Exercise Differences

**Figure 1.**
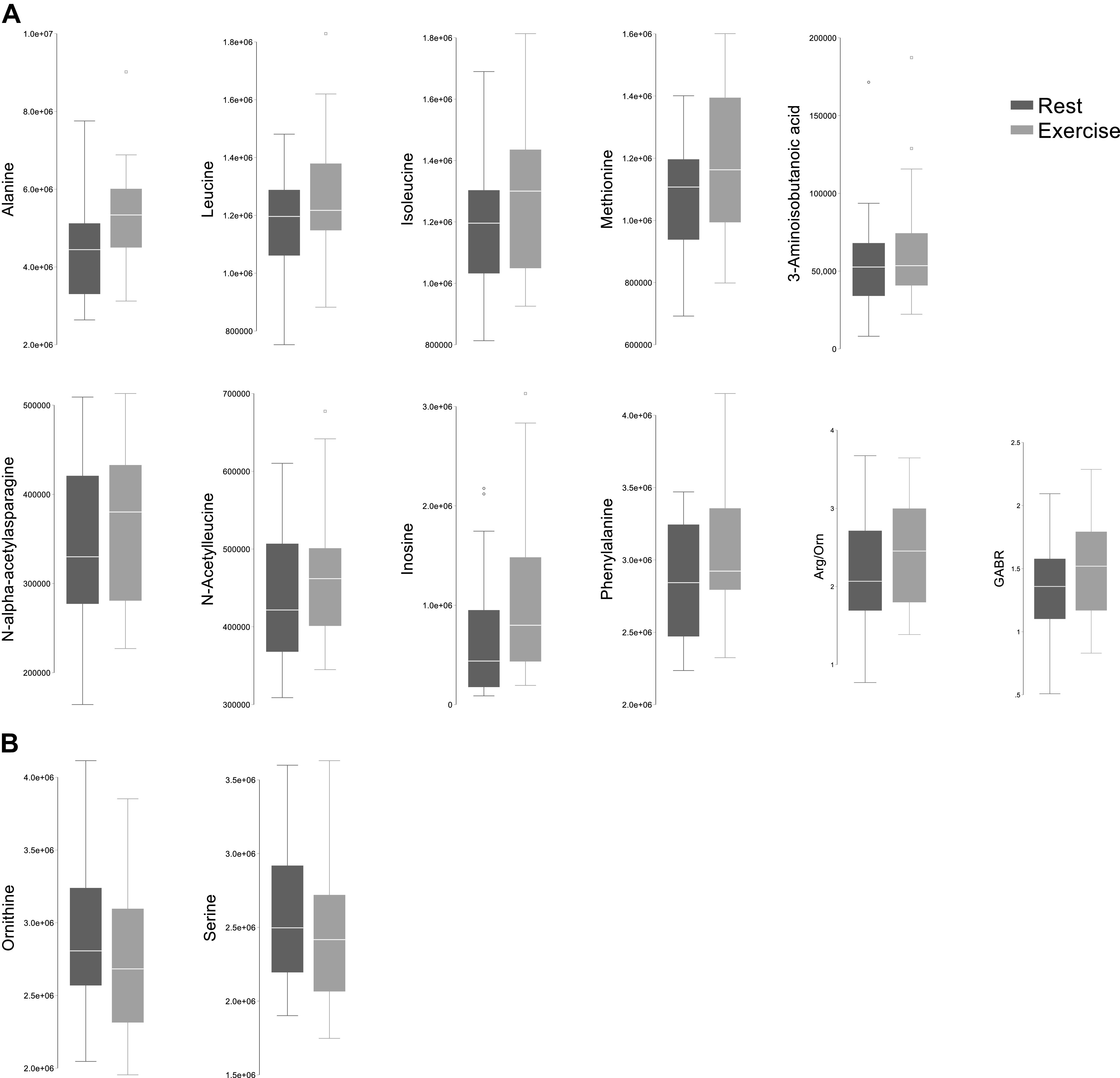
Box plots depicting abundance of metabolites that significantly increase (*A*) or decrease (*B*) postexercise compared with pre-exercise. The center line denotes the median value (50th percentile) and upper and lower hinges denote 75th and 25th percentiles of data, respectively. Upper and lower fences denote 1.5 times the 75th percentile and 25th percentile, respectively. Individual data points beyond upper and lower fences are presented as dots.

In subjects with PAH, 13 metabolite features had significantly different circulating concentrations with exercise compared with rest measurements (Table 2 and Fig. 1). Alanine and the branched chain amino acids (BCAAs) leucine and isoleucine increased in concentration with exercise, along with phenylalanine, a catecholamine precursor; inosine, a precursor of nucleotide synthesis; and N-acetylated forms of leucine and asparagine. Measures of arginine bioavailability, including arg/orn and GABR, increased with exercise, whereas ornithine, a product of the urea cycle, decreased.

**Table 2.** Significant paired rest-exercise differences in metabolite abundance

Metabolite Feature	Class	Pathway	Fold-Change (FC)	log2 (FC)	*P* Value
Alanine	Amino acid	Alanine and aspartate metabolism	1.1936	0.2553	0.0004
Arg/Orn	Amino acid	Urea cycle	1.1909	0.2520	0.0006
Leucine	Amino acid	BCAA metabolism	1.0886	0.1225	0.0035
GABR	Amino acid	Urea cycle	1.1699	0.2264	0.0043
Ornithine	Amino acid	Urea cycle	0.9259	−0.1111	0.0067
Methionine	Amino acid	Methionine, cysteine, SAM and taurine metabolism	1.0891	0.1231	0.0135
N-α-Acetylasparagine	Amino acid	Alanine and aspartate metabolism	1.1272	0.1727	0.0163
Isoleucine	Amino acid	BCAA metabolism	1.0731	0.1018	0.0214
3-Aminoisobutanoic acid	Amino acid	Pyrimidine metabolism	1.1605	0.2147	0.0254
Serine	Amino acid	Glycine, serine and threonine metabolism	0.9452	−0.0814	0.0384
Inosine	Nucleotide	Purine metabolism	1.7256	0.7871	0.0415
N-Acetylleucine	Amino acid	BCAA metabolism	1.0845	0.1171	0.0415
Phenylalanine	Amino acid	Phenylalanine metabolism	1.0560	0.07861	0.0484

BCAA, branched chain amino acid.

There were significant associations between increases in circulating BCAAs with exercise and clinical variables reflective of greater disease severity in PAH. For all ventriculo-arterial parameters, delta metabolite associations are provided in Supplemental Table S1. Greater delta valine was associated with higher PVR at rest; greater delta leucine was associated with higher mPAP both at rest and with exercise. Increases in arginine bioavailability with exercise were associated with higher PVR and lower CO with exercise, though not with rest hemodynamics.

Exercise-induced increases in circulating uridine, a pyrimidine nucleoside found only in RNA (not present in DNA) that is essential for flux through the pentose phosphate pathway, were associated with multiple cardiopulmonary measurements, including adverse exercise hemodynamics (higher mPAP, PVR, and lower CO with exercise), worse RV diastolic function (lower τ), and steeper pulmonary pressure-flow relationships.

### Rest Metabolite Associations

**Figure 2.**
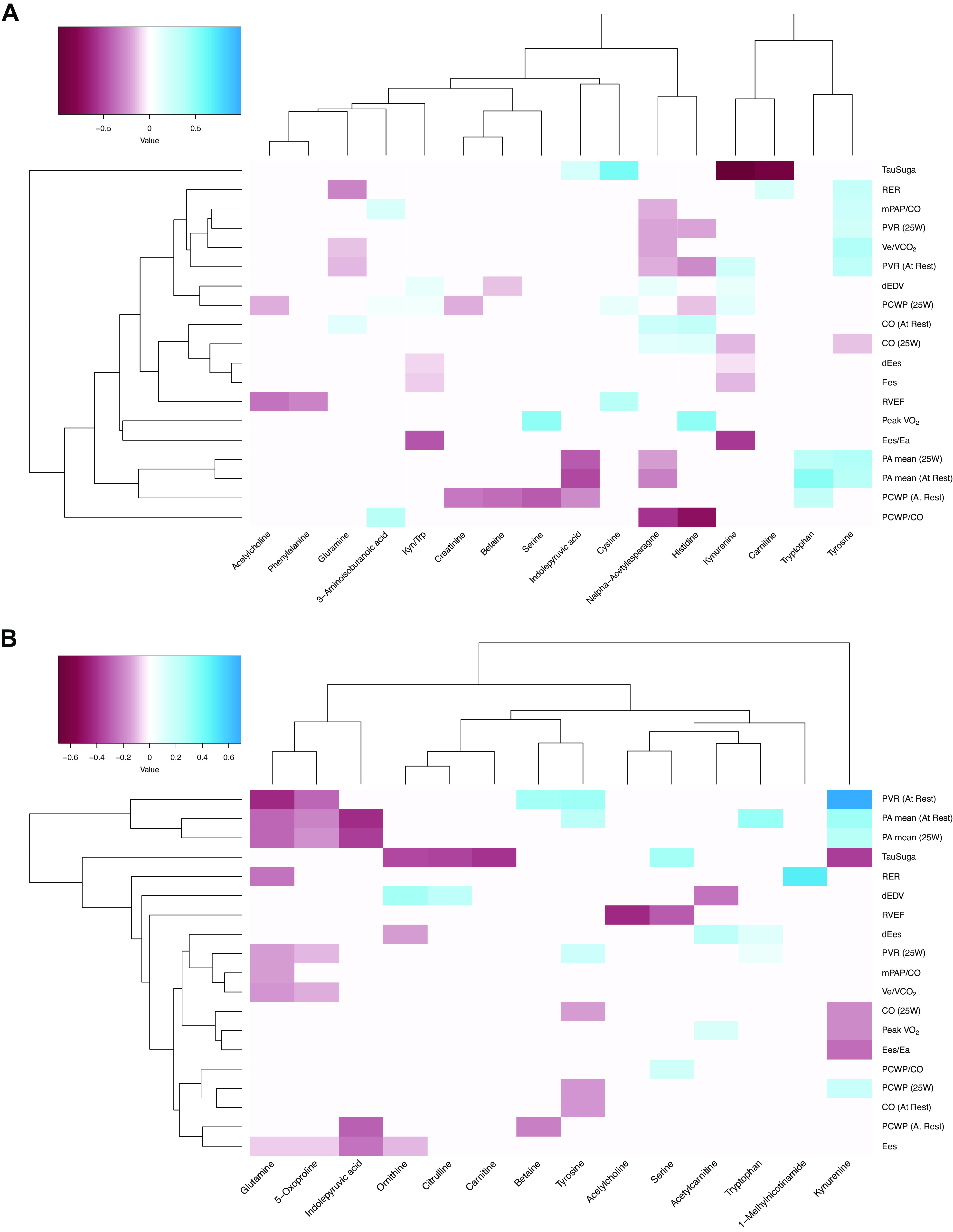
Heatmaps depicting the top 25 unadjusted rest (*A*) and exercise (*B*) metabolite associations with ventriculo-arterial parameters. Parameters are listed along the *y*-axis, and individual metabolites are shown on the *x*-axis. The red-blue color scale represents the value of the *z*-scored coefficient, which reflects the mean standard deviation change for each ventriculo-arterial parameter per one standard deviation increase in metabolite. Red colors correspond to lower values, and blue colors correspond to higher values, as per the color key. Dendrograms reflect hierarchical clustering that orders rows and columns by similarity of associations. CO, cardiac output; Ea, end-arterial elastance; Ees, end-systolic elastance; meanPAP, mean pulmonary arterial pressure; PVR, pulmonary vascular resistance; RER, respiratory equivalence ratio; RVEF, right ventricular ejection fraction; V̇o_2peak_, maximum oxygen uptake.

Metabolic features of the kynurenine pathway, the major route for tryptophan catabolism in humans, were inversely associated with several important measures of RV function, including Ees, Ees/Ea, and RV functional reserve (delta Ees) in vivo. All pre-exercise metabolite associations with clinical parameters are provided in Supplemental Table S2, and a heatmap of the top 25 rest metabolite-phenotype associations is presented in Fig. 2*A*. Rest kynurenine was robustly associated with τ, a measure of RV diastolic function, such that each standard deviation increase in kynurenine concentration was associated with one standard deviation lower τ (β coefficient −0.99, 95% CI −1.12 to −0.69). In addition to kynurenine itself, its ratio to tryptophan, kyn/trp, a surrogate for kynurenine pathway enzymatic activity, was significantly associated with worse RV-PA coupling (Ees/Ea −0.44, 95% CI −0.41 to −0.05), lower RV contractility (Ees −0.11, 95% CI −0.13 to −0.01), reduced contractility with exercise (dEes −0.080, 95% CI −0.117 to −0.005), and RV dilation with exercise (delta end-diastolic volume, dEDV, 0.105, 95% CI 0.017–0.126). Higher resting kynurenine concentrations were also associated with adverse resting pulmonary hemodynamics (mPAP and PVR).

Conversely, indole pathway metabolism, an alternative route for tryptophan catabolism, was associated with better RV function and lower pulmonary pressures: higher indolepyruvate was significantly associated with better RV diastolic function (τ 0.195, 95% CI 0.094–0.561) and lower mPAP both at rest (−0.479, 95% CI −0.653 to −0.284) and with exercise (−0.423, 95% CI −0.487 to −0.096). Higher resting N-acetylasparagine, one of the metabolites that significantly increased in abundance with exercise, was associated with lower pulmonary pressures and better RV function.

Metabolic features of arginine bioavailability measured at rest predicted exercise hemodynamics and pulmonary pressure-flow relationships with exercise. Although exercise-induced increases in arginine bioavailability were associated with higher exercise PVR and steeper multipoint mPAP/CO slopes, greater arginine bioavailability measured during the rest state predicted lower exercise PVR and less steep multipoint mPAP/CO slopes, indicative of more favorable pulmonary vascular responses to exercise. Importantly, in sensitivity analyses, associations between arginine bioavailability and ventriculo-arterial parameters persisted with adjustment for PDE5 inhibitors.

### Exercise Metabolite Associations

Higher kynurenine pathway metabolites measured postexercise were associated with various hemodynamic measures: higher postexercise kynurenine was associated with higher resting RAP (0.298, 95% CI 0.025–0.328), mPAP (0.314, 95% CI 0.045–0.340), and PVR (0.691, 95% CI 0.175–0.930). In general, magnitudes of association for postexercise kynurenine were greater than those for kynurenine measured at rest. Higher kynurenine postexercise was significantly associated with lower V̇o_2max_. All postexercise metabolite associations are shown in Supplemental Table S3, and a heatmap of the top 25 exercise metabolite-phenotype associations is presented in Fig. 2*B*.

Given the known impacts of pulmonary vasodilators on key metabolic pathways, we performed sensitivity analyses adjusting sPLS models for PAH-specific therapies. Heatmaps depicting top metabolite-phenotype associations (at both rest and exercise) are shown in Supplemental Fig. S3, *A* and *B*. Results of these analyses confirmed the robustness of key metabolite-phenotype associations, particularly those involving arginine-NO and kynurenine pathway metabolism.

### Clinically Relevant Dichotomies

**Figure 3.**
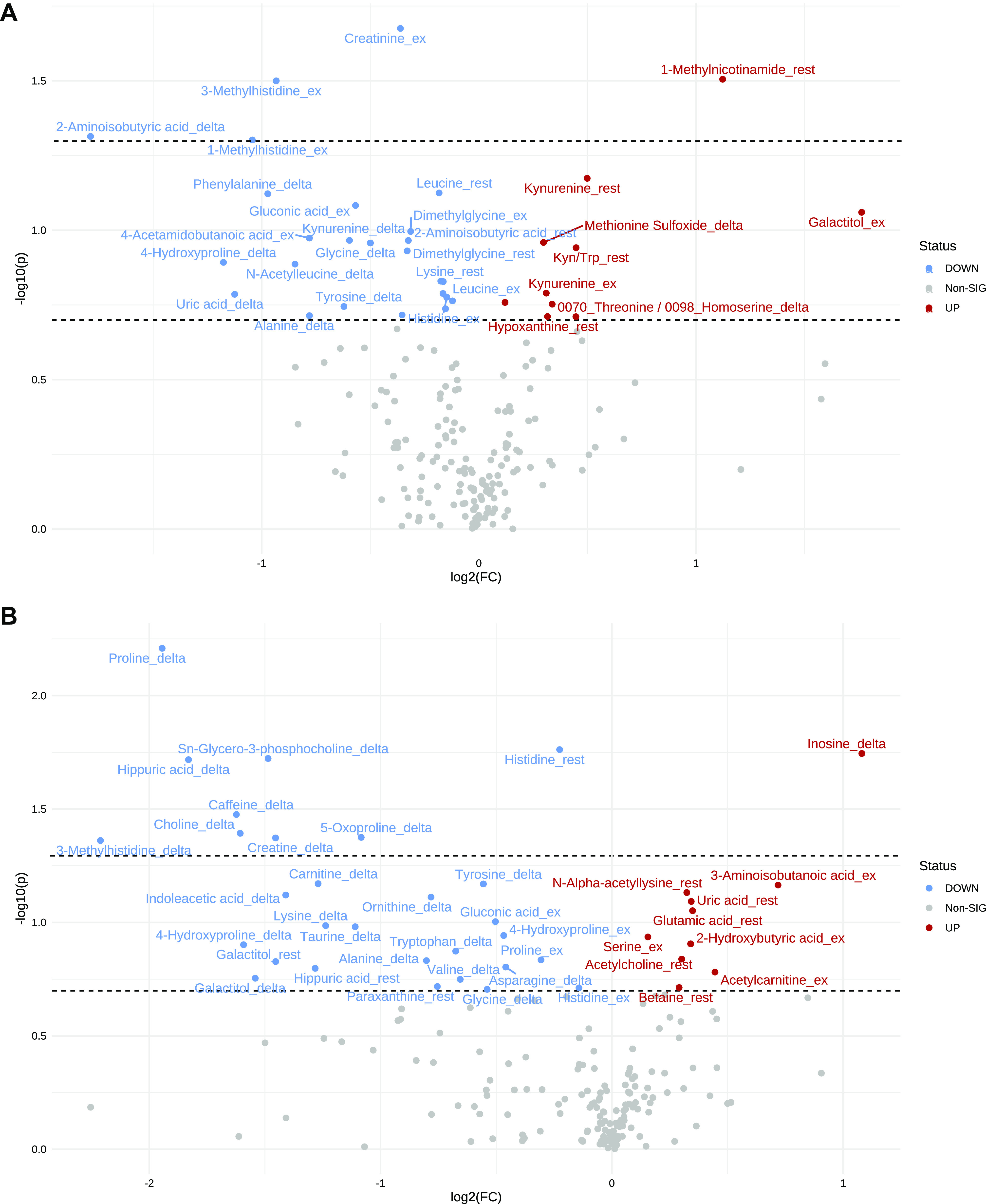
Volcano plots showing the magnitude (*x*-axis) and significance (*y*-axis) of metabolite fold-change differences in subjects with decoupled vs. preserved Ees/Ea (*A*) and with vs. without clinical worsening (*B*). Dots represent individual metabolite features. Features increased in subjects with decoupled Ees/Ea (*A*) or clinical worsening (*B*) are shown with red dots, whereas features decreased are shown with blue dots. The lower horizontal line indicates borderline statistical significance at α = 0.20. The upper horizontal line indicates statistical significance at α = 0.05. Ea, end-arterial elastance; Ees, end-systolic elastance.

To ground our analyses in clinical relevance, we next dichotomized PAH subjects according to whether they possessed *1*) decoupled versus preserved Ees/Ea (as a comprehensive measure of RV functional adaptation), and *2*) did or did not experience a clinical worsening event during the follow-up period. We used a clinically validated cut-point of Ees/Ea <0.65 to signify RV-PA uncoupling (12). Subjects with a coupling ratio <0.65 tended to have higher kynurenine pathway metabolites; higher 1-methylnicotinamide, an NAD metabolite; and higher methionine sulfoxide, a marker of oxidative stress (Fig. 3*A*). Subjects who experienced clinical worsening tended to have higher uric acid, lower histidine, and greater increases in inosine with exercise (Fig. 3*B*).

### Comparisons with NT-proBNP

**Figure 4.**
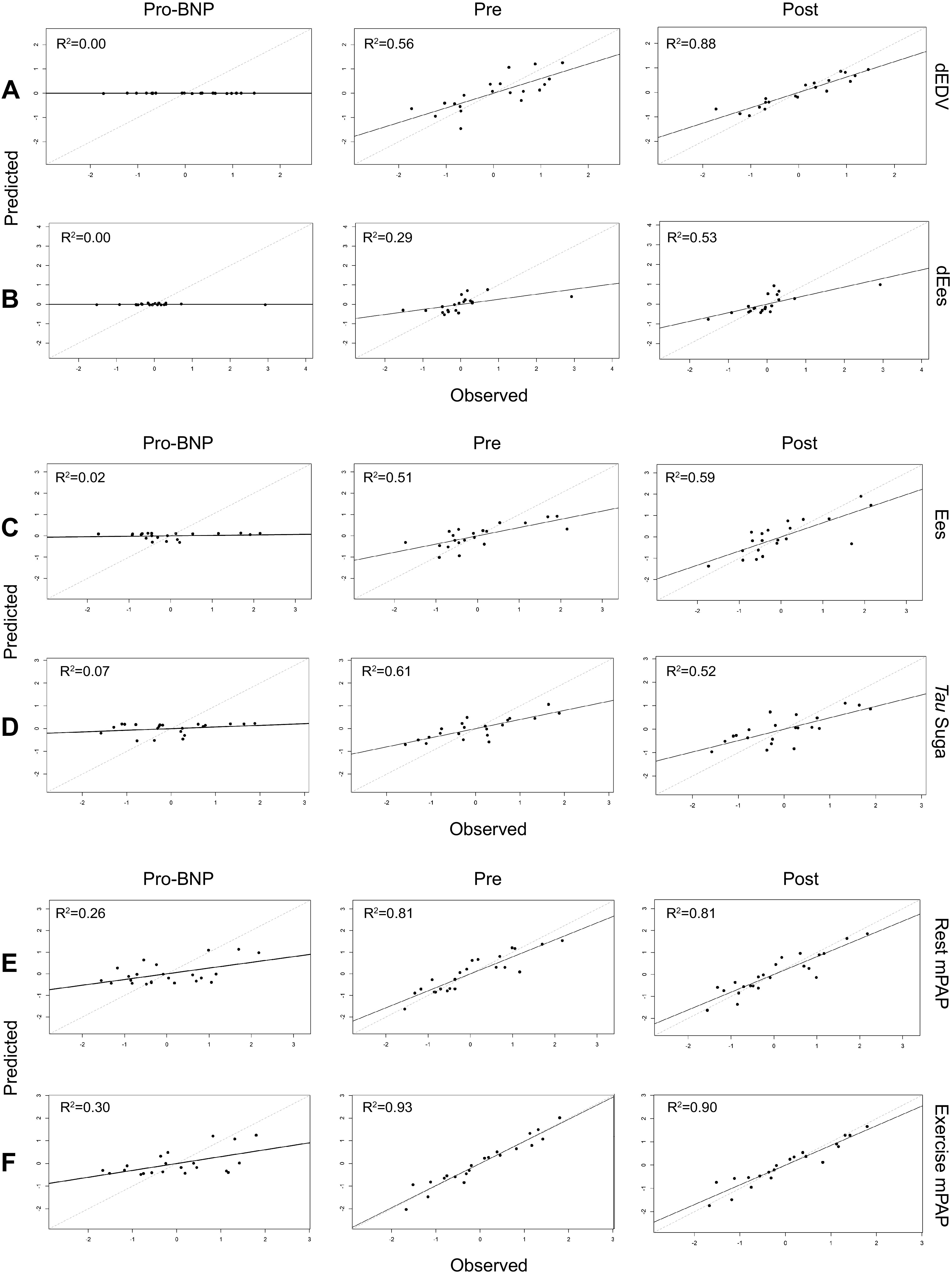
Model fit for sparse PLS models utilizing NT-proBNP, pre-exercise metabolites, and postexercise metabolites as explanatory variables to predict ventriculo-arterial parameters as dependent variables: dEDV and dEes (*A* and *B*); Ees and τ (*C* and *D*); and rest and exercise mPAP (*E* and *F*). Actual values in the data (*x*-axis) are plotted against the values predicted by the models (*y*-axis). *R*^2^ values are rounded to two decimal places. Ees, end-systolic elastance; meanPAP, mean pulmonary arterial pressure; NT-proBNP, N-terminal prohormone of B-type natriuretic peptide; PLS, partial least squares.

The *R*^2^ statistic was used to evaluate the accuracy of metabolite models, compared with NT-proBNP, for predicting ventriculo-arterial parameters. In regression modeling, *R*^2^ describes the proportion of variance in a dependent variable (in this case, a ventriculo-arterial measurement) that is accounted for by an explanatory variable (in this case, selected metabolites or NT-proBNP). Pre- and postexercise metabolites selected by sPLS models outperformed NT-proBNP in predicting RV exercise performance in vivo. NT-proBNP was not informative in explaining variation in dEes (change in RV contractility with exercise) or dEDV (change in RV dilation with exercise) within the cohort (*R*^2^ 0.00). For both dEes and dEDV, postexercise metabolites outperformed rest metabolites: pre-exercise metabolites explained 29% of variance and postexercise metabolites explained 53% of variance for dEes, whereas pre-exercise metabolites explained 56% of variance and postexercise metabolites explained 88% of variance for dEDV (Fig. 4, *A* and *B*). Metabolite combinations for all models depicted in Fig. 4 are shown in Supplemental Tables S1*B*, S2*B*, and S3*B*.

Resting NT-proBNP accounted for the variation present in the relaxation measurement τ very poorly, with only 7% of variance explained, whereas resting metabolites selected by sPLS accounted for 61% of variance in τ, and exercise metabolites accounted for 52% of variance. Similarly, resting NT-proBNP did not explain variation in the load-independent RV contractility metric Ees (*R*^2^ 2%), whereas resting metabolites explained 51% of the variance present, and postexercise metabolites explained 59% of the variance present. Metabolites did not outperform NT-proBNP in accounting for the variation present in the coupling metric Ees/Ea, which relates contractility to afterload (*R*^2^ for NT-proBNP, rest metabolites, and postexercise metabolites 20%, 27%, and 21%, respectively; Fig. 4, *C* and *D*).

Resting NT-proBNP performed better in modeling pulmonary pressures, explaining 26% of the variance in resting mPAP and 30% of the variance in exercise mPAP. However, metabolites selected by sPLS models provided better model accuracy, with rest metabolites explaining 81% of the variance in resting mPAP, and metabolites measured postexercise explaining 90% of the variance in exercise mPAP. Similarly, resting NT-proBNP explained 35% of the variance in resting CO, and 22% of the variance in CO at exercise. Pre-exercise metabolites selected by sPLS models explained 67% of the variance in CO at rest, whereas postexercise metabolites selected by sPLS explained 87% of the variance in exercise CO (Fig. 4, *E* and *F*). Metabolites also outperformed NT-proBNP in explaining variation in PVR at rest and with exercise (Table 3).

**Table 3.** Model accuracy comparisons: proportion of variance for each parameter explained by selected metabolite combinations vs. NT-proBNP

Parameter	Pre-Exercise Metabolite Profile *R*^2^	Postexercise Metabolite Profile *R*^2^	NT-proBNP *R*^2^
Exercise mPAP	0.93	0.90	0.30
Rest PAWP	0.90	0.08	0.06
Rest mPAP	0.81	0.81	0.26
Exercise PVR	0.75	0.61	0.57
Rest PVR	0.74	0.63	0.43
PCWP/CO	0.69	0.74	0.61
Rest CO	0.67	0.58	0.35
TauSuga	0.61	0.52	0.07
RER	0.59	0.39	0.13
dEDV	0.56	0.88	0.00
Ees	0.51	0.59	0.02
Peak V̇o_2_	0.50	0.38	0.18
Ve/V̇o_2_	0.42	0.48	0.29
mPAP/CO	0.37	0.53	0.53
Exercise PAWP	0.37	0.02	0.24
dEes	0.29	0.53	0.00
Ees/Ea	0.27	0.21	0.20
Exercise CO	0.05	0.87	0.22

See Table 1 for abbreviations.

**Figure 5.**
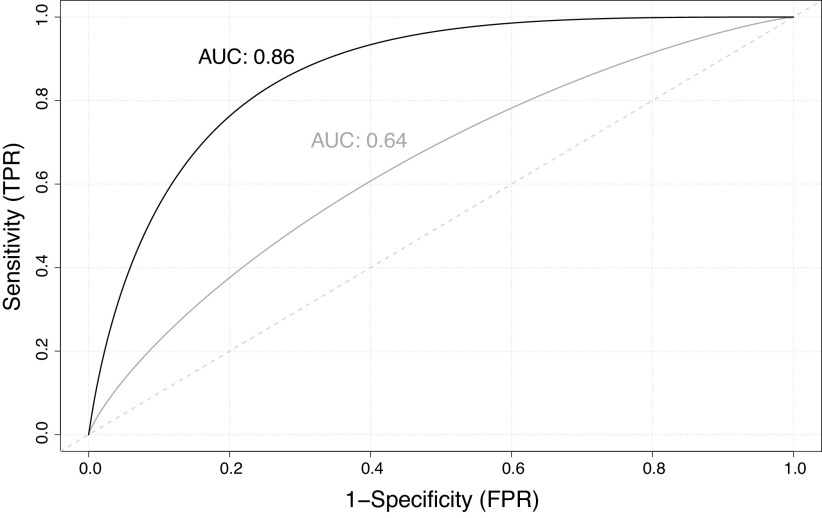
Receiver operating characteristics curves for logistic regression models of clinical worsening. Area under the curve for NT-proBNP (gray curve) and metabolites selected by logistic regression (black curve) are shown. Sensitivity (true positive rate) is plotted on the *y*-axis, and 1-specificity (false positive rate) is plotted on the *x*-axis. NT-proBNP, N-terminal prohormone of B-type natriuretic peptide.

Over an average of 6.3 year of observation, 15 patients experienced a clinical worsening event; nine patients died. A combination of lower histidine (OR 3.62, 95% CI 1.11–19.17) and higher uric acid levels (OR 2.12, 95% CI 0.65–9.43) was associated with greater odds of experiencing clinical worsening, and this combination outperformed NT-proBNP for predicting clinical worsening in the cohort (AUC 0.84 for metabolites vs. 0.64 for NT-proBNP; Fig. 5).

### Pathway Analysis

**Figure 6.**
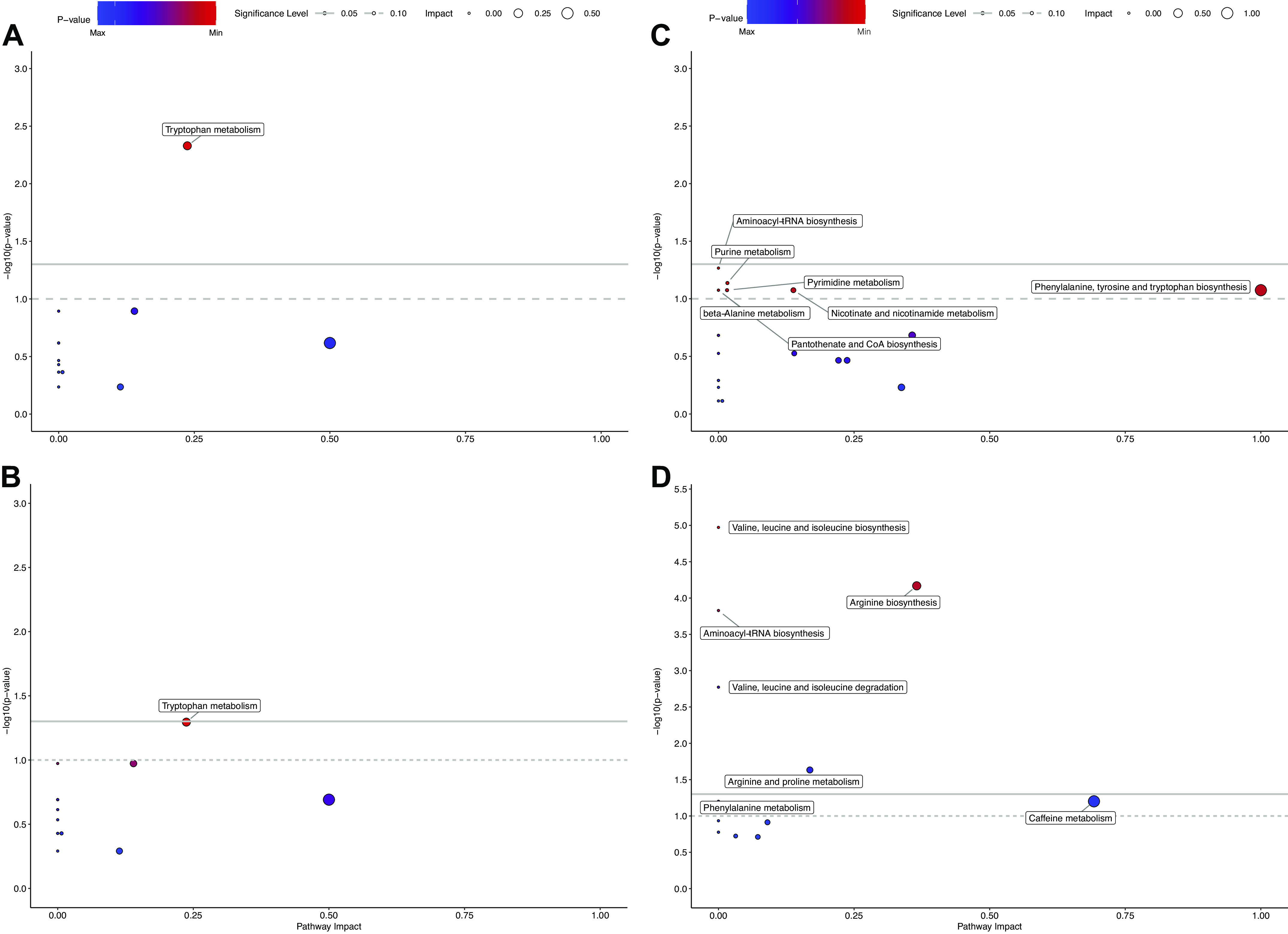
Pathway enrichment and topology analysis for sPLS models with *R*^2^ greater than 80%: rest metabolite prediction of rest mPAP (*A*), exercise metabolite prediction of exercise mPAP (*B*), rest metabolite prediction of rest PCWP (*C*), and exercise metabolite prediction of dEDV with exercise (*D*).Pathway impact is plotted on the *x*-axis, and significance is plotted on the *x*-axis. Point sizes are proportionate to pathway impact. Point colors reflect *P* values from largest (blue) to smallest (red). The KEGG pathway database was used as a reference metabolome. The solid horizontal line indicates statistical significance at α = 0.05. The dashed horizontal line indicates statistical significance at α = 0.10. KEGG, Kyoto Encyclopedia of Genes and Genomes; PCWP, pulmonary capillary wedge pressure; sPLS, sparse partial least squares regression.

The plots in Fig. 6 depict metabolic pathway analysis for models that explained a high proportion of variability (*R*^2^ >80%) for our hemodynamic and RV functional variables of interest. Metabolites that explained a high proportion of variation in mPAP at rest (*R*^2^ 81%) and with exercise (*R*^2^ 90%) were enriched for over-represented tryptophan metabolism, with relatively large pathway impact scores >0.1 (Fig. 6*A*). Metabolic pathways significantly over-represented in other highly predictive models (e.g., for CO and change in RV dilation with exercise) included arginine biosynthesis and metabolism, BCAA biosynthesis and degradation, purine and pyrimidine metabolism, and aminoacyl-tRNA biosynthesis (Fig. 6*B*).

## DISCUSSION

To the best of our knowledge, this study represents the first investigation of metabolomic associations with comprehensive RV functional measurements only obtainable via multibeat RV PV loop analysis, allowing identification of metabolite profiles associated with RV adaptation to increasing afterload, measures of intrinsic RV function such as relaxation and contractility, and measures of RV exercise performance in vivo. Our findings show that tryptophan metabolism is linked with multiple measures of intrinsic RV function, with robust inverse relationships existing between kynurenine and RV diastolic function and kynurenine and RV-PA coupling. Our findings also point to the importance of arginine bioavailability in the cardiopulmonary unit’s response to the stress of exercise. In most instances, metabolite profiles selected by sPLS models outperformed NT-proBNP, particularly for the prediction of measures that are load-independent or reflect the performance of the cardiopulmonary system under stress.

Aberrant tryptophan metabolism was implicated by our metabolomic pathway analyses, and kynurenine pathway metabolites were more accurate than NT-proBNP in predicting pulmonary pressures in our PAH cohort. These results add to a growing body of both clinical and preclinical evidence implicating the kynurenine pathway of tryptophan metabolism as relevant to PAH pathobiology. Lewis et al. (9) identified strong associations between tryptophan metabolites, including kynurenine, and adverse hemodynamics in human subjects with RV-pulmonary vascular dysfunction. More recently, Cai et al. (22) demonstrated kynurenine pathway metabolites are associated with survival and with response to therapy in PAH. Preclinical data suggest kynurenine pathway metabolism may have cardiac-specific effects: in mice, simulation of myocardial infarction (MI) by left coronary ligation induces generation of kynurenine via indoleamine 2, 3-dioxygenase (IDO), an enzyme that catalyzes conversion of tryptophan to kynurenine (23). After MI, genetic deletion of endothelial IDO limited cardiac injury, resulting in improved cardiomyocyte contractility and less adverse ventricular remodeling (23). Conversely, kynurenine supplementation precipitated cardiomyocyte apoptosis (23). Taken together, these observations localize kynurenine pathway metabolism to the cardiopulmonary circuit.

In our cohort, arginine bioavailability proved dynamic with exercise and appeared important to adaptive hemodynamic responses and pulmonary pressure-flow relationships. Higher resting arginine bioavailability was associated with a more favorable hemodynamic profile. With exercise, subjects with more severe hemodynamics augmented arginine bioavailability to a greater extent than subjects with more favorable hemodynamics, suggesting that such augmentation may be compensatory. Arginine is the substrate for synthesis of nitric oxide (NO), which is crucial to vascular homeostasis and effects vasodilation. Patients with PAH and other forms of pulmonary hypertension have reduced arginine bioavailability compared with healthy controls (24, 25), and arginine conversion to urea (via arginase) is known to be inversely associated with mPAP measurements (24). Moreover, NO production from arginine by vascular endothelium in PAH is compromised by inactivated endothelial NO synthase in pulmonary artery endothelial cells (26). Compensatory increases in arginine bioavailability with exertion might function as a counterbalance to these known deficits.

Prior work has suggested that distinct arginine metabolic endotypes exist in PAH, such that some patients have high arginase activity and decreased NO synthesis, whereas others have low arginase activity (24). Relationships between clinical phenotypes and endogenous arginine biosynthesis have not been similarly studied in PAH. However, our results lend credence to small clinical studies that have previously demonstrated improvements in exercise performance with l-arginine supplementation. One small proof-of-concept study demonstrated improvements in 6-min walking distance, V̇o_2max_, and heart rate recovery when subjects with PAH adhered to a prescribed light exercise regimen along with l-arginine supplementation (6,000 mg/day; 27). Another small randomized placebo-controlled trial showed improvements in V̇o_2max_ and reductions in mPAP and PVR in precapillary pulmonary hypertension patients randomized to l-arginine supplementation (28).

Purine and pyrimidine-modified nucleosides and other metabolites have been previously associated with phenotypes and outcomes in PAH (9, 10), and we redemonstrate this in the present study. Uric acid has been associated with survival in both IPAH (29) and SSc-PAH (30) and is a predictor of clinical worsening in the current study. In our cohort, inosine levels dynamically increased with exercise, and exercise-induced increases in uridine were associated with adverse hemodynamics and RV function. It remains unclear whether over-represented purine/pyrimidine metabolism represents hyperproliferation and increased cell turnover in disease, abnormal pentose phosphate metabolism, or, as other authors have postulated, posttranslational modification of tRNAs required for translation of disease-specific proteins (10). Our pathway analyses, which implicate aminoacyl-tRNA biosynthesis in metabolite profiles that robustly predict exercise responses, align best with the latter hypothesis.

In our cohort, increased circulating BCAAs with exercise were associated with more severe PAH. Although increased alanine concentrations are generally observed with exercise, increases in BCAAs with exercise are not demonstrated in healthy subjects. In a systematic review and meta-analysis of 27 human exercise metabolomics studies, leucine and isoleucine concentrations in the blood significantly decreased within 30 min of a bout of exercise, in contrast to our results in PAH (31). One early investigation of myocardial amino acid metabolism following cardiac surgery detected increased net uptake of BCAAs and glutamate postoperatively that was directly correlated with myocardial oxygen consumption (32). BCAAs are elevated in the myocardium of mice and humans with heart failure, and BCAA catabolic defects have been demonstrated (33). Supplementation of BCAAs has been shown to improve ventricular contractility in the failing mouse heart (34). In the context of these prior studies, our results add to a collection of observations suggesting a mismatch between myocardial AA availability and utilization may contribute to experimental and human heart failure. The provisioning of increased AA during stress states, as seen in postischemic cardiac surgery patients and in our patients with PAH, might serve an adaptive function, though this is speculative. Future mechanistic work is needed to clarify the cellular sources and fates of AAs that increase or decrease with exercise in PAH.

In addition to offering pathobiologic insights, our findings underscore the potential for select metabolites to function as disease-specific biomarkers. Metabolite combinations outperformed NT-proBNP, the current clinical gold standard marker, for predicting most hemodynamic and RV functional variables. Improvements in model accuracy were most robust for prediction of variables associated with intrinsic RV function, such as relaxation and contractility, and RV exercise performance. Kynurenine pathway features were among those consistently selected into metabolite models that improved predictive accuracy. Further research is needed to validate selected metabolites as RV-specific biomarkers, including studies that absolutely quantify metabolite abundance and examine statistical discrimination rigorously, but these initial results suggest that the identification of molecules that are pathobiologically related to disease-specific variables may result in improved biomarker calibration.

This study has important limitations, including its modest sample size and lack of a suitable validation cohort. These limitations, though, are inherent to a study design that leverages difficult to perform RV PV loops in subjects with a rare disease. PV loop analysis is a strength of the study, allowing examination of relationships between varied aspects of metabolism and comprehensive RV function. SSc-PAH subjects predominate within our cohort, which reflects referral patterns at our center. Because we are under-powered to examine subtype-specific metabolite associations, we cannot be certain that the associations in our cohort generalize to all PAH. Finally, although we are able to demonstrate novel associations with these analyses, future studies are needed to elucidate the mechanistic functions of metabolic pathways implicated here.

In conclusion, specific metabolite profiles predict various aspects of RV-PA function. Future work is needed to conduct broader-based metabolic profiling in larger, phenotypically rich cohorts, and to integrate metabolite profiles with other—omics layers. Such profiling has the potential to deepen our pathobiologic understanding of PAH, identify targetable pathways, and inform discovery of biomarkers that report on RV-centric features of disease.

## DATA AVAILABILITY

Data will be made available upon reasonable request.

## SUPPLEMENTAL DATA

10.6084/m9.figshare.22263364 Supplemental Tables S1–S3 and Figs. S1–S3: https://doi.org/10.6084/m9.figshare.22263364.

## GRANTS

This work was supported by NIH/NHLBI K23HL153781 (to C.E.S.), R01HL114910 (to P.M.H.), U01HL125175-03S1 (to P.M.H. and S.C.M.), R01HL132153 (to R.L.D. and P.M.H.), K08HL132055 (to K.S.), K23HL146889 (to S.H.). New Investigator Award from the Scleroderma Foundation (to C.E.S.).

## DISCLOSURES

R. J. Tedford reports general disclosures to include consulting relationships with Medtronic, Abbott, Aria CV Inc., Acceleron/Merck, Alleviant, CareDx, Cytokinetics, Itamar, Edwards LifeSciences, Eidos Therapeutics, Lexicon Pharmaceuticals, and Gradient. R. J. Tedford is the national principal investigator for the RIGHT-FLOW clinical trial (Edwards), serves on steering committee for Merck, Edwards, and Abbott as well as a research advisory board for Abiomed. He also does hemodynamic core laboratory work for Merck. S. C. Mathai reports fees from Actelion, United Therapeutics, Janssen, MSD, and Clinical Viewpoints, has served on an Advisory Board for Bayer, and reports a leadership/fiduciary role with the Patient Centered Outcomes Research Institute, all unrelated to the current work. P. M. Hassoun serves on a scientific steering board for MSD, an activity unrelated to the current work. None of the other authors has any conflicts of interest, financial or otherwise, to disclose. 

## AUTHOR CONTRIBUTIONS

C.E.S., S.A., D.G., P.M.H., and R.L.D. conceived and designed research; R.J.T., S.H., R.H., A.R., and M.K. performed experiments; C.E.S. and E.K.G. analyzed data; C.E.S., J.C., S.H., R.H., A.R., S.A., D.G., K.S., R.J.T., T.M.K., S.C.M., P.M.H., and R.L.D. interpreted results of experiments; C.E.S., J.C., and E.K.G. prepared figures; C.E.S. and J.C. drafted manuscript; S.H., E.K.G., R.H., A.R., S.A., D.G., M.K., K.S., R.J.T., T.M.K., S.C.M., P.M.H., and R.L.D. edited and revised manuscript; C.E.S., J.C., S.H., E.K.G., R.H., A.R., S.A., D.G., M.K., K.S., R.J.T., T.M.K., S.C.M., P.M.H., and R.L.D. approved final version of manuscript.
